# Supplementary material for: Learning curve and surgical outcome of robotic assisted colorectal surgery with ERAS program
Source: Sci Rep. 2022 Nov 29;12:20566. doi: 10.1038/s41598-022-24665-w (PMC9709162; doi:10.1038/s41598-022-24665-w)
Supplement: Supplementary file 2 — Supplementary Information 2. [file 41598_2022_24665_MOESM2_ESM.zip › RAL Raw data-Ñ[▒K/098/HS_ñw╝╨░O▒Kñσ.pdf]

正式摘要(電子簽章：000000221047234)		
W95 / W95　☐英文標題(注意:無英文姓名-請補病人基本檔!)	☐【讀取】憑證	

臺中榮民總醫院
出院病歷摘要

轉入號:　　　　轉出號:

[redacted]

**(8)**流水編號	
**(9)**入院日期	110/07/14 (2021)
**(10)**轉科日期	
**(11)**出院日期	110/07/20 (2021)　住院天數計 6 日

診斷

- 入院 :1. GIST at right pelvis, s/p trasvaginal excision on 2020/10/29, under neoadjuvant target therapy with Gilvec
  2. Angiomyolipoma, left kidney, 0.9cm
  3. MVP, diagnosed in 2018 at CV OPD
- 出院 :1. GIST at right pelvis, s/p trasvaginal excision on 2020/10/29, under neoadjuvant with Gilvec, s/p Robotic wedge resection of rectum and vagina + Bilateral D-J + Loop ileostomy on 2021/07/15, s/p removal of bilateral D-J catheter on 2021/07/19
  2. Angiomyolipoma, left kidney, 0.9cm
  3. MVP, diagnosed in 2018 at CV OPD

主訴

- Scheduled operation for rectal tumor post immunotherapy

病史

- This 40-year-old woman has past history of:
  1. GIST at right pelvis, s/p trasvaginal excision on 2020/10/29, under neoadjuvant target therapy with Gilvec
  2. Angiomyolipoma, left kidney, 0.9cm
  3. MVP, diagnosed in 2018 at CV OPD

  She presented with midcycle spotting/browning staining for 3 months, and came to our GYN OPD in 2020/10. PE showed a firm and elastic mass about 2x4x2cm from right pelvic wall between M/3 vaginal and rectal canal. Pelvic CT was arranged on 2020/10/02, reporting a 3.2cm poor enhancing mass at right presacral region of pelvic wall adjacent to rectum; sarcoma or enlarge lymph node or nature to be determined. She then received trasvaginal excision on 2020/10/29, with pathology reporting GIST.

  He was then refered to CRS OPD. Anoscopy in 2020/11 found a firm 2cm tumor at right side of L/3 rectum. Neoadjuvant Gilvec then surgery was suggested. Follow-up abdominal CT on 2021/02/08 reported rectal GIST without obvious change in size. MRI on 2021/06/10 revealed a 25x19x23mm nodular lesion over right perirectal region, compatible with GIST, stable in size.

  Under the impression of GIST at right pelvis, post neoadjuvant with Gilvec for 6 months, she was admitted for Robotic LAR for wide excision including vagina scheduled on 2021/07/15.

體檢發現

-
  - General appearance :
    - easy looking , conscious clear , without cardiopulmonary distress
    - Vital signs自動帶入date 體溫(BT) 脈搏(P) 呼吸(R) 血壓(BP) 身高(BH) 體重(BW) BMI
      20210714 37.1℃ 101次/分 18次/分 111/60mmHg 155cm 41.1kg 17.1
    - Vital signs: B.P. 111/60 mmHg , P.R. 101 /min , R.R. 18 /min , B.T. 37.1 ℃
  - HEENT :
    - Eyes: no anemic conjunctiva, no icteric sclera, normal EOM
    - ENT: no otorrhea, no rhinorrhea, no erythematous mucosa, no injected tonsil, no deformity
  - Neck: supple, freely movable, trachea midline,no deformity, no engorged jugular

- vein , no palpable thyroid or lymph node enlargement
- Chest:
  - Inspection: Grossly normal, symmetric and fully expansion, no accessory muscle used, no operation scar, no spider angiomata, no gynecomastia
  - Palpation: no palpable mass or nodule, no subcutaneous emphysema, no tenderness
  - Ausculation: clear breathing sound
- Heart:
  - Palpation: no thrill, no heave
  - Percussion: normal heart size
  - Ausculation: regular heart beat, normal S1, S2, no gallop, no murmur
- Extremities: No deformity, full ranged of motion without limitation, no clubbing finger or nail cyanosis, no pitting edema, no ulcer or pigmentation, no obvious skin lesion, no varicose vein, no bone, joint or muscle tenderness

CRS CONDITION

- Karnofsky performance status: (100) Normal, no complaints, no evidence of disease
- PE of Abdomen:
- CEA: nil ng/ml
- Imaging Studies:
- Colonoscope report: Anoscopy in 2020/11 found a firm 2cm tumor at right side of L/3 rectum.
- Sono , CT of abdomen and chest Films:Follow-up abdominal CT on 2021/02/08 reported rectal GIST without obvious change in size. MRI on 2021/06/10 revealed a 25x19x23mm nodular lesion over right perirectal region, compatible with GIST, stable in size.
- Figures:
  1. colon & rectum

手術日期及方法

- 手術日期及方法20210715 1. Robot (Xi) assisted wedge resection of the rectum and the vagina
  2. Intraoperative colonoscopy : (-).
  3. High ligation (-). IMA was not approached.
  4. Take down from the splenic flexure : (-)
  5. No Robotic endoGIA was applied.
  6. Proximal cut end or distal cut end : Could not be evaluated
  7. A 12 Fr. penrose drainage tube was inserted from LLQ of abdomen into pelvic area
  8. ADHESION BARRIER FILM was placed below the fascia of the central umbilicus wound
  20210715 Ureter, Double J insertion Ureter, Double J insertion
  20210719 Ureter, Removal of Double J catheter

住院治療經過

- After operation with Robotic LAR for wide excision including vagina + Bilateral D-J insertion on 2021/07/15, the patient was under post-operative care and pain control under PCA use. Hematuria was noted after operation, with blood clots. The patient tolerated clear liquid diet and then Bland No.II diet since 7/17, without N/V, abdominal pain or discomfort. The wound was clean and dry, without infectious signs. Bilateral D-J catheter and Foley's catheter were removed on 7/19. Under relative stable condition, the patient was discharge with OPD follow up.

合併症

一般檢查

- 累積報告 :

DATE	TIME	ALB	TP	BIL.T	BIL.D	ALKP	AST	ALT	LDH	AMY	LIPASE	NH3	Ferritin	UA
1100716	0851	3.5		0.6	0.2		21	10						

DATE	TIME	WBC	RBC	HGB	HCT	MCV	PLT	BLST	NEUT	BAND	SEG	LYM	MON	EOS	BAS
1100716	0916	8440		8.8	25.4	102.0	159		81.9			11.6	5.9	0.2	0.4
1100719	0856	3830		8.9	25.8	100.4	156		57.4			26.4	9.1	6.8	0.3

DATE	TIME	NA	K	CL	BUN	CREAT	eGFR	CA	Mg	P	CRP	ESR	PCT	D-Dimer	LACTATE
1100716	0843														8.6
1100716	0851	143	3.3		9	0.84	79.81	7.9							
1100719	0851	141	3.6		12	0.84	79.81	8.2			4.195				
1100719	0907												0.041		

DATE	TIME	SP-GR	PH	PROT	GLUC	KETON	BILI	UROB	OB	NITR	LEU	RBC	WBC	EPCEL
1100716	1637	1.023	5.5	2+	Negative	Trace	Negative	0.2	3+	Negative	1+	2+	35-50	0-1

DATE	TIME	PH	BEB	SCVO2	PO2	HCO3	HGB	PCO2	TCO2
1100716	0842	7.416	1.2	87.3	52.3	25.8		41.1	27.1

特殊檢查

病理報告

其它

出院時情況

- 3.改門診治療

出院指示

藥名	劑量	劑量單位	用法	頻次	數量	數量單位	現況	天數	單位
$Biofermin tab	2	TAB	PO	TID	42	TAB	TH	7	天
Mag. Oxide tab	2	TAB	PO	BID	28	TAB	TH	7	天
TramTOR cap 50mg	0.5	CAP	PO	Q6H	14	CAP	TH	7	天
Econ tab 40mg	1	TAB	PO	QID	28	TAB	TH	7	天
MOSAD tab 5mg	1	TAB	PO	TID	21	TAB	TH	7	天
(□)Takepron OD tab 30mg	1	TAB	PO	QDAC	7	TAB	TH	7	天
Flurbiprofen 100mg	1	TAB	PO	BID	14	TAB	TH	7	天

出院計劃

- 門診日期
  - date on 2021/07/27

主治醫師_2062F 蔣鋒帆 / 住院醫師_2345B 陳映晴

正式摘要**(電子簽章：000000221047234)**

W95 / W95

doctorRank:B
